# The Health Status of Horses Used for at Least Six Complete Cycles of Loxoscelic Antivenom Production

**DOI:** 10.3390/toxins15100589

**Published:** 2023-09-26

**Authors:** Ana Luísa Soares de Miranda, Bruno Cesar Antunes, João Carlos Minozzo, Sabrina de Almeida Lima, Ana Flávia Machado Botelho, Marco Túlio Gomes Campos, Carlos Chávez-Olórtegui, Benito Soto-Blanco

**Affiliations:** 1 Department of Veterinary Clinics and Surgery, Veterinary College, Universidade Federal de Minas Gerais (UFMG), Av. Antônio Carlos 6627, Belo Horizonte 30123-970, MG, Brazil; analuisa.miranda@hotmail.com (A.L.S.d.M.); camposmtg@gmail.com (M.T.G.C.); 2 Department of Health of the State of Paraná, Centro de Produção e Pesquisa de Imunobiológicos (CPPI), Rua Piquiri 170, Piraquara 80230-140, PR, Brazil; bruno.antunes@sesa.pr.gov.br (B.C.A.); joao.minozzo@sesa.pr.gov.br (J.C.M.); 3 Department of Biochemistry and Immunology, Institute of Biological Sciences, Universidade Federal de Minas Gerais (UFMG), Av. Antônio Carlos 6627, Belo Horizonte 31270-901, MG, Brazil; sabrina.lima79@yahoo.com.br (S.d.A.L.); olortegi@icb.ufmg.br (C.C.-O.); 4 Department of Veterinary Medicine, Veterinary College, Universidade Federal de Goiás (UFG), Campus Samambaia, Goiânia 74690-900, GO, Brazil; anafmb@ufg.br

**Keywords:** antisera production, brown spider, dermonecrosis, loxoscelism, safety evaluation

## Abstract

Antivenom production against *Loxosceles* venom relies on horses being immunized and bled for plasma harvest. One horse can partake in several cycles of antivenom production, which will require years of constant venom and adjuvant inoculation and bleeding. The actual impact on the health of horses that participate in several antivenom-producing cycles is unknown. Therefore, this study aimed to evaluate the general health status of horses that underwent at least six cycles of loxoscelic antivenom production. Seven crossbred horses that had partaken in six to eight complete antivenom-producing cycles were used and established as the immunized group (IG). Under the same handling and general management, eleven horses were established as the control group (CG). The horses were evaluated regarding their general clinical status and had their blood sampled, and an ECG recorded. The IG presented lower RBC and PCV, despite keeping values within inferior limits for the species. Renal function was not impaired, and liver-related enzymes were higher than those in the CG, probably due to liver exertion from immunoglobulin synthesis. ECG showed some abnormalities in the IG, such as atrioventricular block and a wandering atrial pacemaker, corroborated by an increase in CK-MB. The cardiovascular abnormalities were mainly found in the horses that participated in several antivenom-producing cycles. The overall results indicate that these horses had some impairment of their general health status. Once available, some alternative, less toxic antigens should replace the venom for immunization of horses used for antivenom production.

## 1. Introduction

Antivenom remains the most suitable treatment for loxoscelism, a syndrome triggered by the bite from a brown spider (*Loxosceles* spp.) [1,2,3]. The most common manifestation of loxoscelism is cutaneous, and consists of a progressive dermonecrotic lesion with gravitational spreading. To a lesser extent, loxoscelism can manifest as a visceral cutaneous form consisting of systemic hemolysis, intravascular coagulation, and acute renal failure, often leading to death [4,5,6,7]. The venom produced by *Loxosceles* spiders is a mixture of several toxins. Sphingomyelinases D are considered the main toxins of the venom; these enzymes improve inflammation and local tissue injury by cleaving tissue phospholipids [8,9]. Other important toxins are hyaluronidases, which enhance the spread of venom toxins [10]; astacin-like metalloproteases, which hydrolyze the extracellular matrix [11]; and translationally controlled tumor proteins and *Loxosceles* allergen-like toxins, which trigger the release of histamine [12,13]. *Loxosceles* venom contains several other toxins of almost unknown toxic action [9].

Antivenom production is based on immunizing animals, harvesting their blood, purifying it, and procuring an immunobiological solution filled with immunoglobulins to counteract and neutralize the harmful effects of envenomation [14,15,16,17,18,19]. Despite its obvious public health importance, antivenom procurement faces several hindrances, ranging from venom extraction to animal welfare [16,17,20,21], especially in Brazil, where antivenom in loxoscelism is primarily employed [2,22]. Animal welfare discussions focus mainly on horses because they are the primary species used for antivenom procurement [14,18,21,23,24]. Hyperimmunization and multiple immunization protocols to produce *Loxosceles* antivenom can have several adverse effects on horses, such as ulcers, abscesses, and renal failure [21,25]. However, no further studies have focused on the actual clinical impact on the horses’ health, not only regarding the systemic effects of the venom but also on the impact of partaking in several consecutive immunization protocols, which consist of animals undergoing systematic bleeding, physical restraint, and stress.

Therefore, this study aimed to evaluate the general health status of horses used in six or more immunization cycles for loxoscelic antivenom procurement, comparing clinical, hematological, and electrocardiographic findings with those of non-immunized horses.

## 2. Results

### 2.1. Clinical Findings

**Figure 1:**
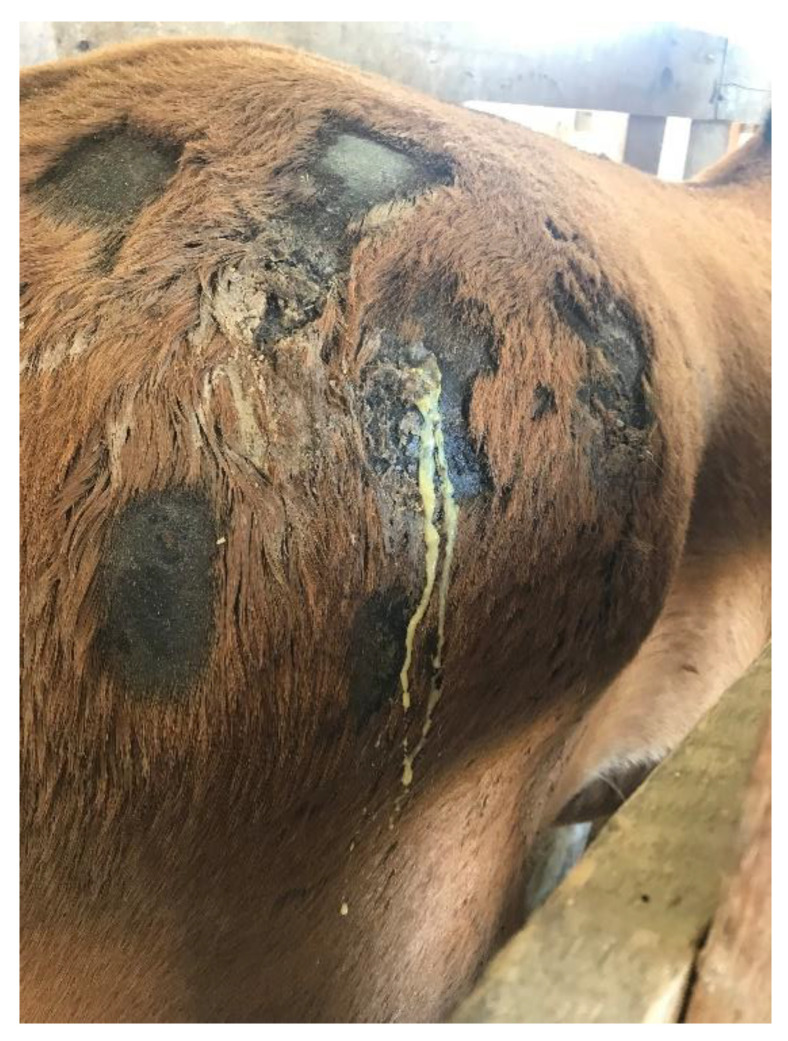
Multiple subcutaneous abscesses formed at infiltration sites in a horse used to produce *Loxosceles* antivenom.

All horses presented satisfactory clinical conditions because no significant clinical alterations were diagnosed. Some horses from the CG, mainly colts and fillies, required a twitch to ensure the handler’s safety. This restraining method might explain why horses from the CG, despite their lack of statistical significance, presented higher heart rates (HR) (56.0 ± 3.80 bpm) than those from the IG (45.9 ± 5.81 bpm). Multiple subcutaneous abscesses (Figure 1) and bilateral thrombophlebitis were observed in the IG horses but not in the controls. Furthermore, all horses from the IG presented with lymphadenopathy. Mandibular, parotid, retropharyngeal, superficial cervical, and ischiatic lymph nodes were palpable, with increased temperature and slight sensibility. No horse from the CG presented any lymph node enlargement.

### 2.2. Electrocardiographic Findings

**Figure 2:**
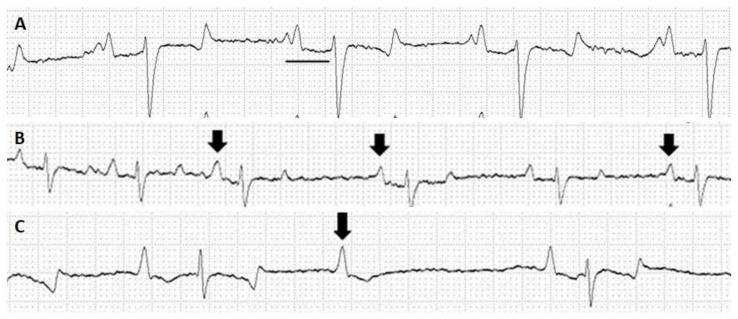
ECG records from horses who had undergone from six to eight immunization cycles for *Loxosceles* antivenom production. (**A**) First-degree AV block. Note the increased PR interval (0.35 s) (black line). (**B**) Wandering atrial pacemaker. Notice the different P-wave morphology (arrows). (**C**) Second-degree AV block. Note that a P wave is not followed by a QRS complex (arrow) despite the maintenance of PP intervals. Recordings of the second deviation of the frontal plane (DII), with a velocity of 25 mm/s and amplitude 1 N.

All horses in the CG presented normal sinus rhythm. Of the seven horses of the IG, six presented some conduction abnormalities, such as first-degree AV block (Figure 2A) (42.9%), polymorphic ventricular tachycardia (14.29%), wandering atrial pacemaker (14.3%) (Figure 2B), second-degree AV block (14.3%) (Figure 2C), sinus tachycardia (14.3%), and sinus arrhythmia (14.3%).

Results from the ECG recordings are presented in Table 1. Only QT intervals and T waves showed statistical significance. The QT interval was higher in the IG horses than in the CG horses, whereas T waves showed higher values in the CG horses.

### 2.3. Hematologic Analyses

Results from the CBC are presented in Table 2. The IG horses showed significantly lower values of RBC, and higher values of MCV, RDW-SD, RDW-CV, and P-LCR, than the CG horses.

### 2.4. Biochemical Analyses

The main blood biochemical parameters obtained between the experimental groups are presented in Table 3. ALT, AST, GGT, and ALP showed statistical significance between experimental groups, with the IG presenting the highest values but within reference ranges for the species. Albumin and triglyceride levels were higher in the CG horses than in the IG horses. Horses from the IG showed CK activities of 386.4 ± 65.2 U/L (reference: 90.0–270.0 U/L), and CK-MB activities of 318.2 ± 60.5 U/L (reference: 5.0–25.0 U/L).

## 3. Discussion

### 3.1. Clinical Findings

The significant clinical changes observed in immunized horses were the presence of multiple subcutaneous abscesses, bilateral thrombophlebitis, and lymphadenopathy. None of these alterations were seen in the CG because no horses had been in contact with adjuvants, venom, inoculations, or bleeding procedures. Subcutaneous abscesses have already been described in horses that underwent the immunization protocol using *Loxosceles* venom [21], and occurred mainly due to the use of adjuvants and the inflammatory response caused by the immunization protocol [26]. Similarly, thrombophlebitis occurred because of systematic venipunctures on the external jugular vein that horses from the IG had undergone [27,28,29,30].

All horses from the IG presented with lymphadenopathy, whereas none of the horses from the CG presented with any lymph node enlargement. Lymphadenopathy is notable in horses with a general inflammatory process [31,32]. Lymph nodes are clusters of germinal cells within a connective tissue framework, containing lymphocytes and macrophages in proximity, which play a vital role in the immune response. Inflammation, such as that incited by the adjuvants and venom used in the present study, can cause lymphadenopathy due to lymphocyte proliferation and accumulation of antigenic materials [33].

### 3.2. Electrocardiographic Findings

*L. intermedia* venom has a direct cardiotoxic effect in isolated, perfused heart preparations and ventricular cardiac myocytes [34]. Thus, the heart function of the horses in this study was evaluated via ECG. The ECG findings in the horses from this study were first-degree AV block in three horses (42.9%), polymorphic ventricular tachycardia, wandering atrial pacemaker, second-degree AV block, sinus tachycardia, and sinus arrhythmia in a horse (14.3%). These ECG abnormalities do not affect the horses’ performance [35,36]. First-degree AV block is a common finding in horses’ ECG because of the higher vagal tone of the species, and can disappear after exercise [31]. This conduction abnormality is diagnosed when the PR interval exceeds 0.2 s; therefore, the conduction from the sinus node to the ventricles is prolonged [35]. Clinical significance, however, is noted only when the PR intervals are significantly prolonged (by over 0.5 s) [37,38]. First-degree AV block was the main finding in the ECGs of this study because it encompassed over 42% of the animals evaluated. Despite the PR increase (but all under 0.5 s), no clinical significance of this finding was highlighted, corroborating its possible physiological origin.

Second-degree AV block is also frequent in clinically healthy horses at rest [37,38,39,40], which is attributed to fluctuations in the autonomic tone, as horses naturally exhibit a high vagal tone [31]. Wandering atrial pacemakers can be caused by a higher vagal tone, a physiological finding that has been previously described by several authors [36,39,41,42]. Sinus arrhythmia can usually be diagnosed in horses at rest because of their high vagal tone. Sinus tachycardia, if not accompanied by other clinical findings, might be related to excitement due to handling or stress [31,35,39].

Polymorphic ventricular tachycardia is defined by four or more consecutive ventricular beatings and can be indicative of primary myocardial disease, hypoxia, or electrolyte imbalance [42,43], and was described in rattlesnake envenoming [44]. Clinical signs of congestive heart failure should be observed because they become more severe over time because of shorter cycle lengths, higher heart rates, and polymorphic ventricular rhythm. Polymorphic ventricular tachycardia is associated with increased electrical inhomogeneity and instability, thus increasing the risk of developing a fatal rhythm [45,46,47].

The QT intervals and T waves showed statistical significance. The QT interval represents cardiac repolarization, and an increase was observed in the IG compared with the CG. The QT might be influenced to some extent by HR, body weight, sex, autonomic tone, and environment. Horses may present a considerable interbreed variation for this variable [48], which explains the difference observed in this study. All horses were crossbreeds, and the CG comprised younger horses. Despite this statistical difference, it is essential to highlight that both experimental groups maintained QT values within the reference range for the species, and no relevant clinical finding was correlated with this parameter variation. It is important to emphasize that an increase in the QT interval predicts ventricular arrhythmias [48,49,50]; therefore, animals showing this finding must be monitored. Regarding T waves, higher values were observed in the CG horses. The T wave might be correlated to hyperkalemia or myocarditis, but only when extreme values (>1.4 mV) are observed, which was not the case in the present study [37,40].

### 3.3. Hematologic Analyses

Despite remaining within the reference values of the species, the IG horses maintained hematological variables such as RBC and PCV on inferior limits. Age can be a relevant factor that can influence these parameters, and horses from the IG evaluated here were 15 years old or older, whereas those of the CG ranged from 5 to 8 years old. The reduction in the RBC count with a compensatory increase in MCV in older horses is probably due to the impaired regenerative capacity of the bone marrow of geriatric horses [51,52]. In this study, MCV was superior in the IG compared with the CG. Thus, these horses might present some anemia, perhaps due to partaking in numerous and systematic bleedings for antivenom procurement, as well as an impaired regenerative bone marrow capacity.

A slight neutrophilia pattern was observed in the IG, possibly due to the several immunization protocols these horses had undergone, with the effects of adjuvants and venom toxicity [21]. Neutrophils were largely recruited in acute inflammation [53], so thrombophlebitis and subcutaneous abscesses in the IG horses might contribute to this discrete neutrophilia.

### 3.4. Biochemical Analyses

All enzymes related to liver function (ALT, AST, GGT, and ALP) showed statistically higher values in the IG horses, but these values were within reference ranges for the species. Horses used for antivenom production have a higher hepatic demand because the liver is responsible for globulin synthesis [54]. However, only severe increases exceeding the upper reference limits of these enzymes indicate a dysfunction or impairment [53], which was not the case. Similar results were obtained in horses used for snake antivenom production [18]. On the other hand, no apparent kidney impairment occurred in the horses in the present study, in which urea and creatinine were kept within reference ranges and had no statistical significance between the IG and the CG. This finding is similar to that of horses used for snake antivenom production [18].

IG horses presented significantly lower albumin levels than the CG. A decrease in albumin levels (but maintenance within physiological ranges) in horses used for antivenom production has been previously described [18,54]. A decrease in albumin production might be correlated to inflammation [55], a reaction to which horses of the IG have systematically been exposed. The induction of cytokines, especially IL1, IL6, and TNF-α, acts on the liver to produce new acute phase proteins, such as serum amyloid A and fibrinogen [55].

Triglycerides were also significantly higher in the CG horses, and some animals in this group presented triglycerides above reference values for the species. These horses presented higher body scores and weights than IG horses and had visible adiposity deposits, such as neck crests, which might explain their higher triglyceride levels and a higher cholesterol tendency [56].

The measurement of CK-MB is used to detect myocardial injuries (CK-MB) [57]. Horses in the IG showed CK activities of 386.4 ± 65.2 U/L (reference: 90.0–270.0 U/L) and CK-MB activities of 318.2 ± 60.5 U/L (reference: 5.0–25.0 U/L). Because CK-MB is a fraction of total CK, a CK increase might be correlated solely with the CK-MB increase because of the extremely high levels of CK-MB presented here.

Increased CK-MB can be caused by myocardial injury [58,59], although some studies have shown that an increase in CK-MB does not reflect in a histological lesion and comes from a transitory increase in sarcolemma permeability [60,61]. In this study, bleeding for antivenom production may have caused transient ischemia, and thus caused the myocardial injury reflected in the ECG alterations and CK-MB elevation. In addition to bleeding, some toxins of the *Loxosceles* venom could exert cardiotoxicity. This venom caused impairment of cardiac function due to calcium flow disruption and its increased intracellular concentration [34], which might explain the high level of rhythm abnormalities in the ECG recordings and increased CK-MB observed in the present study.

### 3.5. Limitations and Perspectives

All horses used in this study were at the end of their use for loxoscelic antivenom production and would no longer be used for this purpose. These horses were used for six to eight complete immunization cycles. This variation in the number of cycles was due to individual antibody titration responses against the venom; therefore, it was necessary to repeat the immunization when the titer was insufficient to produce the antivenom. The ideal would be to be able to monitor the animals throughout the entire period of use as producers of antibodies against the venom, with another group of the same number of animals kept under the same conditions to serve as a control. Despite the limitations of the present study, it was possible to verify that horses subjected to six to eight complete loxoscelic immunization protocols showed some impairment in their general health status. To improve the welfare of horses, a less toxic antigen should replace the venom for immunization.

As using venom for the immunization of horses was responsible for damage to their health, one suggestion is to use non-toxic antigens that can induce the formation of antibodies that neutralize the *Loxosceles* venom toxins. Some recombinant toxins, especially sphingomyelinases isoforms, were synthesized and induced the production of neutralizing antibodies, but these recombinant toxins were not entirely non-toxic to the immunized animals [2]. Thus, some chimeric proteins containing multiepitopes for *Loxosceles* toxins were developed to immunize animals without causing major health damage while efficiently producing neutralizing antibodies [62,63]. However, the use of animals may, in the future, be replaced using in vitro laboratory methods to produce large amounts of neutralizing antibodies. Currently, the most viable strategy for obtaining specific neutralizing antibodies for *Loxosceles* antivenom is their production via hybridoma cell cultures [64]. Hybridomas are formed by spleen B cells of immunized animals fused to myeloma cells [65]. This technology has the advantage of not using animals for antibody production, but it is not yet fully developed for commercial antivenom production.

## 4. Conclusions

Horses used in six to eight loxoscelic immunization protocols showed multiple subcutaneous abscesses, bilateral thrombophlebitis, lymphadenopathy, and cardiovascular abnormalities. Biochemical analyses detected no significant kidney or liver dysfunction. Overall, the results indicate that these horses had some impairment of their general health status. Once available, alternative, less toxic antigens should replace the venom used for immunization of horses for antivenom production.

## 5. Materials and Methods

### 5.1. Animals and Experimental Groups

The institutional Ethical Committee for the Use of Animals of the Federal University of Minas Gerais approved the animal study protocol (protocol code 159/2019, approved on 4 June 2018).

Eighteen healthy crossbred horses, both male and female, weighing 501.86 ± 95.29 Kg and 18.86 ± 3.34 years of age, were used. Horses were housed in the Centro de Produção e Pesquisa de Imunobiológicos (CPPI—Center of Production and Research of Immunobiologicals), Piraquara, Paraná, Brazil. Horses were kept at pasture and were fed 6 kg of alfalfa hay, 2 kg of hydrated/germinated oat grains per day, and 2 kg of horse ration containing 12% protein. Water and mineral salt were provided ad libitum. Horses were vaccinated yearly against encephalomyelitis, influenza, leptospirosis, rabies, strangles, and tetanus. All horses were purchased from certified properties free from infectious equine anemia and glanders.

Seven of these horses underwent six to eight complete immunization cycles for loxoscelic antivenom production and were named the immunized group (IG). These animals were at the end of loxoscelic antivenom production and would no longer be used for this purpose. This variation in the number of immunization cycles was due to individual antibody titration responses against the venom; therefore, it was necessary to repeat the immunization when the titer was insufficient to produce the antivenom. The venom used for the immunization was a pool of venom collected from *L. intermedia*, *L. gaucho*, and *L. laeta* specimens captured within the Paraná and Santa Catarina States, Brazil. These horses’ first immunization protocol was described earlier [21], and the consecutive ones consisted only of reimmunization cycles because hyperimmunization is performed only once during the horses’ lives. When the horses acquired a satisfactory immunoglobulin titrate, bleeding for antivenom industrial procurement was performed. When the horses were evaluated in this experiment, they had not undergone any bleeding or immunization for at least 60 days, and some had already retired from their activities.

For the control group (CG), eleven naïve horses were used. These horses were not used for any immunization protocol but were maintained under the same nutritional and general management as the IG horses. They were ready to start the loxoscelic antivenom production process and were monitored in another study [21].

### 5.2. Clinical, Electrocardiographic, and Hematological Evaluations

All horses were subjected to a thorough physical examination [31,32,66]. Clinical examinations were performed after mechanical restraint without sedation. After that, an ECG evaluation was performed on horses in an orthostatic position in a quiet environment. The ECG recordings were acquired using a portable 12-channel digital electrocardiograph (TEB ECG Vet, Tecnologia Eletrônica Brasileira, São Paulo, SP, Brazil). Alligator metal clips embedded in alcohol were connected to the skin, and the electrodes were fixed. Electrodes were placed according to the Dubois configuration, placing electrodes one and two next to the spine tuberosity of both scapulae; electrode three on the xiphoid process of the sternum; and electrode four on the proximal cranial region of the left forelimb [67]. For recording, the speed was adjusted to 25 mm/s, the sensitivity was set at 1 cm = 1 mV, and bipolar (DI, DII, DIII) and augmented unipolar (aVR, aVL, aVF) leads were recorded. The parameters evaluated were cardiac rhythm, cardiac frequency, P (ms), P (mV), PR, QRS, and QT intervals, R and T waves, and ST segment levels. QRS polarity in the bipolar and unipolar augmented leads was used to determine the electric axis.

Blood samples were collected from the external jugular vein using vacuum tubes containing ethylenediaminetetraacetic acid (EDTA) or a clot activator (BD Vacutainer, Becton Dickinson, Curitiba, PR, Brazil). Whole blood samples collected with EDTA were used for hematological analyses using an automatic cell counter (pocH-100Iv-Diff, Sysmex, São Paulo, SP, Brazil). The parameters measured were red blood cell count (RBC), hemoglobin, packed cell volume (PCV), mean corpuscular volume (MCV), red blood cell distribution width (RDW), white blood cell count (WBC), lymphocytes and the sum of other WBCs, such as neutrophils, monocytes, and basophils (OTH), total platelet count (PLT), mean platelet volume (MPV), platelet distribution width (PDW), and platelet clump (P-LCR).

Serum samples were used for biochemical analyses using automated equipment (Cobas Mira Plus, Roche, Montclair, NJ, EUA). The measured parameters were alanine aminotransferase (ALT), aspartate transaminase (AST), alkaline phosphatase (ALP), gamma-glutamyl transpeptidase (GGT), urea, creatinine, total proteins (TP), albumin, globulins, glucose, amylase, cholesterol, triglyceride, lactate, lactate dehydrogenase (LDH), creatine kinase (CK), and creatine kinase/isoenzyme MB fraction (CK-MB).

### 5.3. Statistical Analysis

Statistical analyses were performed using the R software (version 3.6.1). The normality of data was evaluated using the Shapiro-Wilk test. The Mann–Whitney *U* test was used to compare non-parametric data (hematology, serum albumin and GGT), and Student’s *t*-test was employed to compare parametric data (all other parameters). The level of statistical significance was set at *p* < 0.05.

## Figures and Tables

**Table 1:** ECG results from horses used in several immunization protocols for *Loxosceles* antivenom production (immunized group) and non-immunized horses (control group). Data are presented as mean ± SEM.

ECG Parameter	Control Group	Immunized Horses	Reference Values
Heart rate (bpm)	56.0 ± 3.80	45.9 ± 5.81	28–40
P (ms)	118.8 ± 6.50	133.6 ± 10.9	<160
P (mV)	0.43 ± 0.02	0.43 ± 0.04	
PR (ms)	282.8 ± 20.6	297.0 ± 18.2	<500
QRS (ms)	128.3 ± 6.93	133.7 ± 5.03	<140
R (mV)	1.03 ± 0.15	0.73 ± 0.11	
QT (ms)	434.2 ± 18.8	509.3 ± 23.2 *	<600
T (mV)	0.46 ± 0.04	0.30 ± 0.03 *	

* Significant difference (*p* < 0.05, Student’s *t*-test) from the control group.

**Table 2:** Complete blood count panel of immunized horses using *Loxosceles* spp. venom (immunized group) and non-immunized horses (control group). Data are presented as mean ± SEM.

Parameter	Control Group	Immunized Horses	Reference Values
WBC (cell × 10^3^/μL)	11.4 ± 0.93	9.66 ± 0.83	5.20–13.9
RBC (cell × 10^6^/μL)	8.67 ± 0.48	6.61 ± 0.28 *	6.4–10.0
PCV (%)	39.8 ± 2.49	33.9 ± 1.31	32.0–47.0
MCV (fL)	45.8 ± 0.58	51.4 ± 1.16 *	37.0–59.0
PLT (cell × 10^3^/μL)	114.2 ± 17.4	113.4 ± 10.5	120.0–256.0
Lymphocytes (%)	33.5 ± 2.89	30.1 ± 1.90	
OTHR (%)	66.5 ± 2.89	69.9 ± 1.90	
Eosinophils (%)	0.00 ± 0.00	0.43 ± 0.43	
LYM (cell × 10^3^/μL)	3.68 ± 0.31	3.03 ± 0.23	1.5–7.7
OTHR (cell × 10^3^/μL)	7.75 ± 0.88	6.83 ± 0.75	
Eosinophils (cell × 10^3^/μL)	0.00 ± 0.00	0.04 ± 0.04	
RDW-SD (fL)	38.0 ± 0.61	44.6 ± 1.12 *	
RDW-CV (%)	20.9 ± 0.28	22.3 ± 0.40 *	21.0–25.0
PDW (fL)	9.78 ± 0.09	12.0 ± 1.10	
MPV (fL)	8.23 ± 0.12	9.07 ± 0.38	5.3–7.8
P-LCR (%)	6.70 ± 1.43	17.9 ± 4.46 *	

Red blood cell count (RBC); packed cell volume (PCV); mean corpuscular volume (MCV); white blood cell count (WBC); red blood cell distribution width (RDW); sum of other WBC, such as neutrophils, monocytes, and basophils (OTHR); total platelet count (PLT); platelet distribution width (PDW); mean platelet volume (MPV); platelet clump (P-LCR). * Significant difference (*p* < 0.05, Mann–Whitney *U* test) from the control group.

**Table 3:** Blood biochemical parameters of immunized horses using *Loxosceles* spp. venom (immunized group) and non-immunized horses (control group). Data are presented as mean ± SEM.

Parameter	Control Group	Immunized Horses	Reference Values
Urea (mg/dL)	35.8 ± 1.33	36.3 ± 3.58	21.4–51.5
Creatinine (mg/dL)	1.36 ± 0.11	1.16 ± 0.06	0.4–2.2
ALT (U/L)	4.32 ± 1.07	16.4 ± 2.68 *	3.0–23.0
AST (U/L)	155.3 ± 10.6	223.7 ± 10.9 *	226–336
ALP (U/L)	159.3 ± 12.9	237.5 ± 29.6 *	86.0–295.0
GGT (U/L)	10.4 ± 1.32	24.0 ± 9.22 *	6.0–32.0
Glucose (mg/dL)	101.3 ± 7.97	115.7 ± 11.7	62.0–134.0
TP (g/dL)	8.90 ± 0.46	7.81 ± 0.20	6.0–8.0
Albumin (g/dL)	3.63 ± 0.23	2.18 ± 0.08 *	2.4–4.1
Globulin (g/dL)	5.28 ± 0.35	5.63 ± 0.26	2.6–4.0
Cholesterol (mg/dl)	91.4 ± 5.89	79.3 ± 2.96	75.0–150.0
Triglycerides (mg/dl)	42.9 ± 3.02	19.9 ± 2.86 *	4.0–44.0

Alanine aminotransferase (ALT); aspartate transaminase (AST); alkaline phosphatase (ALP); gamma-glutamyl transpeptidase (GGT); total protein (TP); creatine kinase (CK). * Significant difference (*p* < 0.05, Mann–Whitney *U* test for GGT and albumin, Student’s *t*-test for the other parameters) from the control group.

## Data Availability

The datasets generated for this study are available on request from the corresponding author.
